# Mushroom-derived bioactive components with definite structures in alleviating the pathogenesis of Alzheimer’s disease

**DOI:** 10.3389/fphar.2024.1373660

**Published:** 2024-05-21

**Authors:** Xue Jiang, Yu Song, Changshun Lv, Yinghui Li, Xiangru Feng, Hao Zhang, Yujuan Chen, Qingshuang Wang

**Affiliations:** ^1^ College of Life Science and Technology, Changchun University of Science and Technology, Changchun, China; ^2^ Koch Biotechnology (Beijing) Co., Ltd., Beijing, China

**Keywords:** mushroom, bioactive components, definite structure, Alzheimer’s disease, mechanism

## Abstract

Alzheimer’s disease (AD) is a complicated neurodegenerative condition with two forms: familial and sporadic. The familial presentation is marked by autosomal dominance, typically occurring early in individuals under 65 years of age, while the sporadic presentation is late-onset, occurring in individuals over the age of 65. The majority of AD cases are characterized by late-onset and sporadic. Despite extensive research conducted over several decades, there is a scarcity of effective therapies and strategies. Considering the lack of a cure for AD, it is essential to explore alternative natural substances with higher efficacy and fewer side effects for AD treatment. Bioactive compounds derived from mushrooms have demonstrated significant potential in AD prevention and treatment by different mechanisms such as targeting amyloid formation, tau, cholinesterase dysfunction, oxidative stress, neuroinflammation, neuronal apoptosis, neurotrophic factors, ER stress, excitotoxicity, and mitochondrial dysfunction. These compounds have garnered considerable interest from the academic community owing to their advantages of multi-channel, multi-target, high safety and low toxicity. This review focuses on the various mechanisms involved in the development and progression of AD, presents the regulatory effects of bioactive components with definite structure from mushroom on AD in recent years, highlights the possible intervention pathways of mushroom bioactive components targeting different mechanisms, and discusses the clinical studies, limitations, and future perspectives of mushroom bioactive components in AD prevention and treatment.

## 1 Introduction

Alzheimer’s disease (AD) is a significant neurodegenerative disorder characterized by behavioral abnormalities and cognitive decline in clinical presentation (Koseoglu, 2019). AD, the predominant type of dementia, has the highest prevalence among all dementia cases, encompassing approximately 60%–80% of them. As age advances, the prevalence of AD exhibits an alarming trend, doubling every 5 years after the age of 65 years (Ding et al., 2022). Based on the findings and projections of the World Health Organization, the global population affected by AD is estimated to be approximately 50 million. Importantly, this number is expected to triple, reaching 150 million by 2050 (Zhang et al., 2022). The projected number of individuals diagnosed with AD in China is predicted to surpass 30 million by 2050 (Li et al., 2021a).

Two forms of AD have been identified: one is familial, and the other is sporadic. The familial presentation is characterized by autosomal dominance, with an early onset in individuals under 65 years of age (accounting for 1%–5% of cases), and it involves the alteration of specific genes like the presenilin 1 gene, the presenilin 2 gene and the APP gene. The sporadic presentation is late-onset and occurs in individuals over the age of 65. While age is recognized as the primary risk factor, sporadic AD is a complex condition with other contributing factors, including traumatic brain injury, gender, environmental pollution, depression, social isolation, physical inactivity, metabolic syndrome, low academic level, and genetic predisposition. The majority of AD are characterized by late-onset and sporadic (Andrade-Guerrero et al., 2023). At present, there is an incomplete understanding of the underlying mechanisms of AD, with its primary pathological characteristics involving abnormal phosphorylation of tau protein and excessive accumulation of amyloid beta (Aβ) (Zhang, Wang, et al., 2022b). Multiple hypotheses have been proposed to explain the etiology of AD, encompassing the tau protein hypothesis, amyloid hypothesis, cholinergic hypothesis, oxidative stress (OS) hypothesis, neuroinflammation hypothesis, apoptosis hypothesis, neurotrophic hypothesis, endoplasmic reticulum (ER) stress hypothesis, excitotoxicity hypothesis, and mitochondrial dysfunction hypothesis etc (Liu et al., 2019a; Serrano-Pozo, Das, and Hyman, 2021; Zhang, Wang, et al., 2022b).

Existing pharmacological treatments for AD focus on rectifying imbalances in neurotransmitters, which are believed to arise from the accumulation of tau proteins and impaired neuronal function. The primary objective of these interventions is to restore proper neurotransmission and alleviate cognitive and behavioral symptoms associated with the disease (Zhu et al., 2021). Current drugs approved by the Food and Drug Administration for managing cognitive symptoms of AD include memantine, a glutamate regulator, and acetylcholinesterase inhibitors (AChEIs). Memantine acts by reducing the levels of glutamate, a neurotransmitter believed to contribute to neurotoxicity observed in AD (Atri, 2019). However, research on memantine has yielded ambiguous findings, as some clinical trials have provided limited evidence regarding its efficacy as a treatment for AD (Neal et al., 2021). AChEIs increase the levels of acetylcholine (ACh), a crucial neurotransmitter involved in learning, attention, and memory (Zhu et al., 2021). At present, three AChEIs (galantamine, rivastigmine, and donepezil) have been approved as drugs targeting cholinergic transmission (Khan, Barve, and Kumar 2020). The effectiveness of AChEI drugs in enhancing cognitive function in individuals with AD has been limited, and their use is linked to various adverse effects including vomiting, nausea, diarrhea, syncope, and bradycardia (Perez-Gomez et al., 2021). Considering the debatable efficacy of AChEIs and their associated side effects, the overall risk-benefit relationship of these drugs remains uncertain. In addition to these drugs, there are several other types of medications either in the market or in the clinical stage, such as drugs directed at β1 amyloid (Aducanumab, Verubecestat) and calcium channel blockers (Nimodipine) (Chris Min, 2019; Topcu, 2023; Kaplan et al., 2019). It is important to note that while these drugs are effective in alleviating AD symptoms, they do not possess the capability to prevent or cure the disease itself. The drugs used to treat AD and their associated side effects are shown in Supplementary Table S1. The current medications used for AD treatment primarily target downstream factors, such as neurotransmitter imbalances, rather than directly addressing the accumulation of tau proteins and beta-amyloid, inflammation, and OS, which are hypothesized to drive the progression of AD (Santos, Chaves, and Várnagy, 2021). In addition, these drugs often result in undesirable adverse reactions and side effects. Given the absence of a cure for AD, there is a need to discover alternative natural agents with fewer side effects for AD treatment.

Mushrooms exhibit a wide range of pharmacological properties owing to the presence of various bioactive substances, demonstrating multi-targeted bioactivities with high safety, low toxicity, and affordable cost. Consequently, they have garnered considerable interest from numerous researchers (Patel et al., 2021). Many bioactive components derived from mushrooms, such as carbohydrates, phenols, alkaloids, terpenoids, and lactones, have enormous potential to alleviate AD progression. Therefore, investigating the therapeutic effects of mushroom bioactive substances on AD has become increasingly important (Tong et al., 2023). Among prominent studies on bioactive components derived from mushrooms in the field of neurodegeneration, erinacine A stands out. This compound, extracted from the mycelia of *Hericium erinaceus*, has been extensively investigated for its neuroprotective and neurotrophic properties. Currently, clinical trials are being conducted to assess its potential for alleviating AD symptoms (Li et al., 2020a). Lanostane-type triterpenoids are a group of bioactive mushroom components that specifically target neuro-inflammation. Recent studies have shown that lanostane-type triterpenoids isolated from *Inonotus obliquus* significantly inhibit the production of nitric oxide (NO) and inducible nitric oxide synthase (iNOS) in lipopolysaccharide (LPS)-stimulated BV2 microglial cells (Kou et al., 2021a). Cyathane diterpenoids, another group of bioactive compounds derived from mushrooms, have garnered attention owing to their notable biological activities in AD. Polyoxygenated cyathane diterpenoids derived from *Cyathus africanus* promoted nerve neurite outgrowth induced by nerve growth factor (NGF) in PC12 cells. One of these compounds, allocyathin B2, inhibited the production of NO in LPS-stimulated BV2 cells (Wei et al., 2018). Numerous bioactive substances from mushrooms have been shown to alleviate the pathogenesis of AD in different *in vivo* and *in vitro* models. Therefore, investigation of effective mushroom bioactive components for AD treatment could offer a promising alternative to conventional therapies.

This review provides a comprehensive overview of the underlying mechanisms of AD and presents the latest findings regarding the therapeutic effects of bioactive compounds with definite structures derived from mushrooms. Additionally, the potential of these mushroom-derived bioactive compounds as novel therapeutic strategies for AD treatment is discussed. The aim of this study was to establish a foundation for utilizing these bioactive components of mushrooms as nutraceutical drugs against AD.

## 2 Retrieval strategy

This review extensively examines the existing literature on the beneficial effects of bioactive components from mushrooms in AD. To gather relevant studies, we conducted searches in prominent databases, including NCBI and Web of Science, using specific keywords, such as mushroom, bioactive components, structure, Alzheimer’s disease, hyperphosphorylated tau, amyloid formation, cholinesterase dysfunction, OS, neuroinflammation, neuronal apoptosis, neurotrophic factors (NTFs), ER stress, excitotoxicity, and mitochondrial dysfunction. Articles considered for inclusion were published between 2008 and 2023, and their selection was based on a thorough analysis of titles and abstracts. In addition, we reviewed the reference lists of the identified articles to identify any further relevant studies. This review encompasses related studies conducted on diverse species to ensure a comprehensive understanding of this topic. Non-scientific experiments or review articles were deliberately omitted to maintain focus on rigorous scientific research.

## 3 Mechanisms of AD

AD is an intricate neurodegenerative disorder with progressive progression and insidious onset. Although AD was initially documented over a hundred years ago, there is still no established theory to explain its cause, development, and pathogenesis, nor is there a treatment that can effectively stop the progression of the disease or address its root cause (Silva et al., 2023). However, several hypotheses have been proposed to elucidate the origin of AD, including the Aβ cascade, tau, and cholinergic hypotheses. Diverse mechanisms involved in various biochemical pathways, such as OS, neuroinflammation, neuronal apoptosis, NTFs, ER stress, excitotoxicity, and mitochondrial dysfunction also contribute to neurodegeneration in an additive or synergistic manner, which requires careful consideration in the development of effective AD therapies. The important mechanisms involved in AD are summarized and presented in this section (Supplementary Figure S1), which contributes to the understanding of the etiology of AD.

### 3.1 Aβ cascade hypothesis

Amyloid precursor protein (APP) is a membrane-spanning protein found abundantly in brain tissues. APP can safeguard the nervous system by regulating the intracellular calcium balance and synaptic transmission, which are fundamental for upholding regular physiological functions in the brain (Sanchez et al., 2019). In 1992, John Hardy et al. introduced the Aβ cascade hypothesis, which has since undergone a gradual process of refinement and improvement (Hur, 2022). According to the prevailing perspective of this hypothesis, Aβ is a polypeptide generated through the ongoing hydrolysis of APP under the influence of β secretases and γ secretases (Choi et al., 2023). These Aβ fragments can precipitate and aggregate within the cellular matrix, eventually leading to the formation of senile plaques (Zhang, Lin, et al., 2022). Aβ aggregates exert two primary effects on the central nervous system (CNS), both during and after senile plaque formation. First, they can directly affect nerve cells, leading to neurotoxicity (Zhang et al., 2021). Additionally, Aβ aggregates can activate astrocytes and microglia in the brain (Goedert, 2015). Prolonged and excessive activation of microglia can lead to the phagocytosis of synapses and neurons (Bhatia et al., 2022). Research has indicated that Aβ plays a critical role in the progression of AD; specifically, the soluble oligomers formed by Aβ demonstrate elevated levels of neurotoxicity (Hoshi 2021b).

### 3.2 Tau hypothesis

Tau is a soluble microtubule-associated protein predominantly present in neuronal cells within the brain. Its primary function involves preserving microtubule stability while also contributing to the maintenance of the cytoskeleton and facilitating the assembly of microtubules (Takeda, 2019). In the normal brain, tau protein exhibits low phosphorylation levels. However, in the brains of individuals with AD, tau is hyperphosphorylated. This phosphorylated form of tau aggregates and forms neurofibrillary tangles (NFTs), which subsequently contribute to neuronal loss and impairment in spatial memory (Silva et al., 2019; Takeda, 2019). The formation of NFTs serves as a significant biological indicator of AD pathology, impacting cognitive function and instigating neurodegenerative alterations (Montalto and Ricciarelli, 2023). Furthermore, hyperphosphorylation of tau protein frequently occupies microtubule binding sites, which hinders the binding of kinesin to microtubules and consequently diminishes the amount of kinesins on microtubules (Dixit et al., 2008). This indicates that hyperphosphorylation of the tau protein plays a crucial role in the development of AD. Nevertheless, in both physiological and pathological states, the mechanism of tau phosphorylation is exceedingly complex, which poses substantial difficulties for research. Currently, the numerous mysteries surrounding this process remain unraveled.

### 3.3 Cholinergic hypothesis

The presence of cholinergic deficiency is regarded as an early indication of AD, and cholinergic neurotransmitters play a crucial role in numerous physiological processes within the brain (Nedogreeva et al., 2021). ACh, a vital neurotransmitter in the brain, is crucial for physiological functions such as memory and learning. Research indicates that, with the advancement of AD, cholinergic neurons deteriorate, resulting in the loss of neurotransmitters and subsequent cognitive decline (Liang et al., 2022). Choline acetyltransferase (ChAT) and acetylcholinesterase (AChE) are enzymes responsible for the synthesis and hydrolysis of ACh, respectively, and play significant roles in regulating the levels of ACh (Xia et al., 2022). Hence, modulating the contents of ChAT and AChE to enhance ACh levels represents a viable approach for intervention in the progression of AD.

### 3.4 OS

Research has indicated a close correlation between OS and AD pathogenesis, and OS damage induced by free radicals is considered to contribute to the development of AD (Butterfield and Halliwell, 2019). OS arises from a significant imbalance between the antioxidant defense system and the generation of oxidative components like reactive oxygen species (ROS) (Nolfi-Donegan, Braganza, and Shiva, 2020). Mitochondria are the primary intracellular source of ROS, and excessive ROS accumulation can disrupt mitochondrial homeostasis and function, ultimately leading to apoptosis of neuronal cells (Singh et al., 2019). ROS can also induce the oxidation of intracellular lipids and proteins, resulting in structural and functional impairment of various membrane proteins, thereby exhibiting neurotoxic effects (Scherz-Shouval and Elazar, 2011). Furthermore, studies have demonstrated that ROS can trigger the deposition of phosphorylated tau and aggregation of neuronal Aβ in the brain during the early stages of AD pathogenesis (Merighi et al., 2022). Consequently, antioxidant effects play a significant role in AD treatment.

### 3.5 Neuroinflammation

Neuroinflammation plays a pivotal role in AD pathogenesis. Microglia, as essential immune surveillance cells, are primarily responsible for maintaining microenvironmental homeostasis of cells under normal circumstances (Li et al., 2021). Microglia exhibit two distinct phenotypes: M1 and M2. The M1 phenotype is associated with a pro-inflammatory state, releasing pro-inflammatory factors, whereas the M2 phenotype represents an anti-inflammatory state, releasing mediators that suppress inflammation (Zhang et al., 2022b). Microglia can phagocytize and degrade Aβ during normal physiological functions. However, when microglia undergo abnormal activation, they release inflammatory mediators and neuroinflammatory cytokines, ultimately leading to the initiation and exacerbation of neuroinflammation (Wei et al., 2019). Consequently, an effective approach to intervention in AD involves reducing the production of pro-inflammatory factors by limiting microglial activation and phenotypic transformation.

### 3.6 Neuronal apoptosis

Neuronal apoptosis is a significant contributor to AD development. The pathogenesis of AD involves substantial neuronal loss and is influenced by various factors. As a death protease, Caspase-3 is recognized as a pivotal executioner of apoptosis that facilitates cell death by cleaving molecules associated with DNA repair (Zhang et al., 2022b). Therefore, regulating the activation of Caspase-3 is crucial for preventing neuronal apoptosis. The B-cell lymphoma-2 (Bcl-2) family is instrumental in the regulation of neuronal apoptosis. It comprises Bcl-2 and Bcl-2-Associated X (Bax), which form a balanced system. Increased Bax expression promotes neuronal cell apoptosis, whereas elevated Bcl-2 expression suppresses it. Thus, the Bcl-2/Bax ratio critically influences apoptosis and survival of neuronal cells (Pena-Blanco and Garcia-Saez, 2018b). Neuronal cytochrome C (Cyt-C) primarily exists in the occurrence of OS, and a decrease in the mitochondrial membrane potential (MMP) results in the release of Cyt-C, contributing to neuronal apoptosis (Chen et al., 2019). As a result, regulating neuronal apoptosis is critical for delaying or treating AD progression.

### 3.7 NTFs

NTFs are compact secretory proteins that have a critical effect on growth, survival, differentiation, synaptic plasticity, and myelination of neuronal cells (Nasrolahi et al., 2019). NTFs, such as NGF, brain-derived neurotrophic factor (BDNF), and glial cell-derived neurotrophic factor (GDNF), play a critical role in supporting the survival, maintenance, and regeneration of particular groups of neurons in the brain of adults (Allen et al., 2013). NGF is an essential member of the NTF family that can exert nutritive and potentially protective effects on nerve cells (Mitra, Behbahani, and Eriksdotter, 2019). BDNF, an NTF primarily synthesized within the brain, can bind to receptor tyrosine protein kinase B, leading to the promotion of neurogenesis in the hippocampus, enhancement of synaptic plasticity, and inhibition of neuronal apoptosis (Kowianski et al., 2018; Miranda et al., 2019). GDNF can regulate cholinergic transmission, safeguard dopaminergic neurons, prevent the degeneration of noradrenergic neurons in the locus coeruleus, and provide protection against cognitive impairment in AD (Nasrolahi et al., 2022). Reduced levels of these NTFs have been associated with the symptoms and pathology of the disease, and the use of replacement strategies is being explored as potential therapies for AD.

### 3.8 ER stress

ER stress results from disturbances in the structure and function of the ER, with perturbation of calcium homeostasis and the accumulation of misfolded proteins. The ER response involves alterations in specific proteins, leading to translational attenuation, degradation of misfolded proteins, and the induction of ER chaperones. Exacerbated or prolonged ER stress triggers cellular signals, which ultimately result in cell death. ER stress has been implicated in the development of AD (Choi et al., 2010). Hence, targeting the protective activity against ER stress is a significant objective for the treatment or prevention of AD. The need for novel protective substances has prompted us to screen for the protective activities of bioactive components derived from mushrooms.

### 3.9 Excitotoxicity

Glutamate excitotoxicity was first characterized by John Olney in 1969, and refers to the damage and demise of neurons caused by prolonged or excessive exposure to excitatory amino acids, particularly glutamate (Olney, 1969; Binvignat and Olloquequi, 2020). Glutamate serves as the primary excitatory neurotransmitter within the mammalian central nervous system (CNS), likely constituting one-third of all rapid excitatory synapses in various brain areas including the hippocampus, cerebellum and cortex (Doble, 1999). Glutamate stimulates a multitude of postsynaptic receptors, among which is the N-methyl-D-aspartate (NMDA) receptor, a connection that has been associated with memory and dementia (Liu et al., 2019b). Excitotoxicity takes place under some pathological conditions when decreased uptake and/or increased release of glutamate cause overactivation of its receptors (Binvignat and Olloquequi, 2020). This leads to a sudden massive influx of water, Cl^−^ and Na^+^, causing the neurons to swell and eventually undergo necrosis. Furthermore, the excessive activation triggers unusually high concentrations of intracellular Ca^2+^, which in turn activate catalytic enzymes, produce toxic free radicals, and disrupt cellular energy production. These Ca^2+^-related pathways result in a delayed apoptotic neuronal loss (Choi et al., 1987). Thus, it has been proposed that the glutamatergic pathway is a potential therapeutic target for AD treatment.

### 3.10 Mitochondrial dysfunction

Mitochondria are plastic and mobile organelles primarily tasked with generating adenosine triphosphate (ATP), yet they also play crucial roles in cellular processes, including the biosynthesis of nucleotides, amino acids, and lipids, the modulation of calcium levels, and the participation in autophagy and apoptosis (Leaw et al., 2017). Maintaining neuronal integrity is critically dependent on mitochondrial function due to the high energy demands of neurons. Dysfunction of mitochondria can worsen the condition of AD (Kalani et al., 2023). In AD, mitochondrial dysfunction encompasses reduced ATP production, compromised respiratory complex activity, diminished MMP, impaired Ca^2+^ buffering, hindered axonal mitochondrial transport, and alterations in mitochondrial dynamics (including fusion, fission, and biogenesis). Additionally, there is an increase in ROS production, damage to mitochondrial DNA, and activation of the mitochondrial permeability transition pore. Furthermore, defects occur in the mitochondria–ER interactions that are essential for regulating Ca^2+^ and lipid homeostasis (Fišar, 2022).

## 4 Anti-AD effect of bioactive components with definite structure from mushroom

**FIGURE 1:**
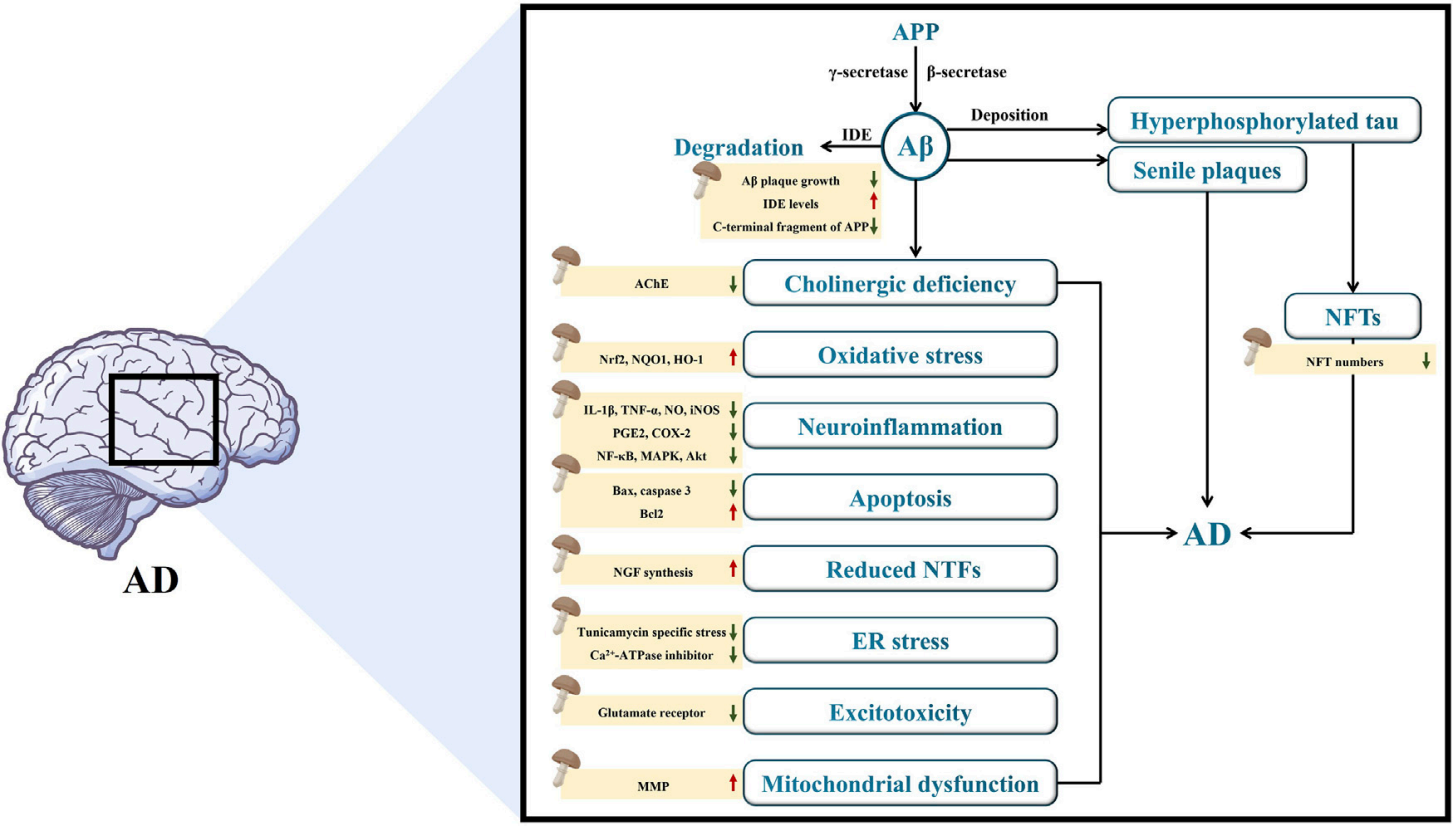
The mechanisms of mushroom bioactive components in alleviating the pathogenesis of AD.

In this section, we primarily explore the protective and/or preventive effects of bioactive components from mushrooms with a definite structure against AD (Figure 1; Table 1). Studies have revealed the involvement of hyperphosphorylated tau, amyloid formation, cholinesterase dysfunction, OS, neuroinflammation, neuronal apoptosis, NTFs, ER stress, excitotoxicity, and mitochondrial dysfunction in AD. These events eventually lead to the occurrence and progression of AD, either separately or in combination. Recent studies have shown that mushroom bioactive components with a definite structure have anti-AD properties and have the ability to improve learning, memory, motor, and cognitive impairment by modulating the aforementioned processes in different *in vitro* and *in vivo* models. According to our review, mushroom bioactive components with definite structures that show anti-AD potential can be classified into several classes, including diterpenoids, triterpenoids, alkaloids, phenolic ingredients, ubiquinone, nucleoside analogs, diterpenoid-xylosides, fatty acid amides, steroids, and quinazoline. In addition, they have the potential to serve as therapeutic agents for AD treatment.

**TABLE 1:** Effects of mushroom-derived bioactive components with definite structure on AD.

Mechanism	Mushroom species	Bioactive component	Molecular weight (kDa)	Chemical formula	Models	Potential mechanism	References
Targeting β-amyloid	*Hericium erinaceus*	Erinacine A	433.27	C_25_H_36_O_6_	APP/PS1 Transgenic Mice	Reduced the load of Aβ plaques in the brain, affects the size and quantity of amyloid core region, inhibiting the growth of the peripheral region of amyloid protein	Chen et al., 2016; Tzeng et al., 2018
		Erinacine S	429.23	C_25_H_34_O_6_	APP/PS1 Transgenic Mice	Reduced the load of Aβ plaques in the brain, affects the size of amyloid core region, inhibiting the growth of the peripheral region of amyloid protein	Chen et al., 2016; Tzeng et al., 2018
					C57BL/6J mice		
	*Antrodia camphorata*	13-epi-3β,19-dihydroxylabda-8 (17),11E-dien-16,15-olide	334.46	C_20_H_30_O_4_	Neonatal cortical neurons in Harlan Sprague Dawley rat offspring	Reduced neurotoxicity induced by Aβ and significantly protected neurons from Aβ damage	Chen et al. (2006)
		3β,19-dihydroxylabda-8 (17),11E-dien-16,15-olide	334.45	C_20_H_30_O_4_	Neonatal cortical neurons in Harlan Sprague Dawley rat offspring	Reduced neurotoxicity induced by Aβ and significantly protected neurons from Aβ damage	Chen et al. (2006)
		19-hydroxylabda-8 (17)-en-16,15-olide	320.47	C_20_H_32_O_3_	Neonatal cortical neurons in Harlan Sprague Dawley rat offspring	Reduced neurotoxicity induced by Aβ and significantly protected neurons from Aβ damage	Chen et al. (2006)
Targeting tau	*Ganoderma lucidum*	Ganoderic acid A	277.00	C_30_H_44_O_7_	APP/PS1 transgenic mice	Reduced the number of NFTs in the cytoplasm	Bao, 2001; Liang et al., 2019; Yu et al., 2020b
		Ganoderic acid B	439.00	C_30_H_44_O_7_	APP/PS1 transgenic mice	Reduced the number of NFTs in the cytoplasm	Bao, 2001; Liang et al., 2019; Yu et al., 2020b
Targeting cholinergic system	*Cortinarius infractus*	10-hydroxy-infractopicrin	277.10	C_17_H_13_N_2_O_2_^+^	AChE from bovine erythrocytes	Inhibited the self-aggregation of Aβ1-40, suppressed AchE activity	Geissler et al. (2010)
		Infractopicrin	261.10	C_17_H_13_N_2_O^+^	AChE from bovine erythrocytes	Inhibited the self-aggregation of Aβ1-40, suppressed AchE activity	Geissler et al. (2010)
Anti-oxidant activities	*Inonotus obliquus*	3,4-dihydroxybenzalacetone	139.04	C_7_H_6_O_3_	PC12 Cells	Reduced the production of intracellular ROS, inhibit the phosphorylation of p38-MAPK pathways	Nakajima, 2007; Nakajima et al., 2009b
	*Antrodia camphorata*	Antroquinonol	413.27	C_24_H_38_O_4_	APP transgenic mice	Reduced oxidative stress by activating the Nrf2 pathway	Lee et al., 2007; Chang, Chen, and Cheng, 2015
	*Ganoderma lucidum*	Ganoderic acid A	277.00	C_30_H_44_O_7_	APP/PS1 transgenic mice	Restored Nrf2, NQO1, and HO-1 antioxidant protein levels	Bao, 2001; Liang et al., 2019; Yu et al., 2020a; Yu et al., 2020b
		Ganoderic acid B	439.00	C_30_H_44_O_7_	APP/PS1 transgenic mice	Restored Nrf2, NQO1, and HO-1 antioxidant protein levels	Bao, 2001; Liang et al., 2019; Yu et al., 2020a; Yu et al., 2020b
Targeting neuroinflammatory	*Cordyceps militaris*	Cordycepin	251.11	C_10_H_13_N_5_O_3_	BV2 microglial cells	Salvaged nerve growth and developmental damage in inflammation-induced hippocampal culture neurons, targets Akt, MAPKs, and NF-κB in microglia and inhibits iNOS, COX-2, and pro-inflammatory cytokine expression	Ahn, 2000; Jeong et al., 2010; Peng et al., 2015
	*Cyathus hookeri*	Cyahookerin B	283.27	C_24_H_37_O_4_	BV2 microglial cells	Significantly inhibited the production of nitric oxide, thereby reducing the occurrence of neuroinflammation	Tang et al. (2019)
Targeting anti-apoptotic	*Inonotus obliquus*	3,4-dihydroxybenzalacetone	139.04	C_7_H_6_O_3_	PC12 Cells	Inhibited of PC12 cell apoptosis by activating Bax and inhibited p38-MAPK	Nakajima, 2007; Nakajima et al., 2009a
	*Ganoderma lucidum*	Ganoderic acid A	277.00	C_30_H_44_O_7_	APP/PS1 transgenic mice	Markedly reduced Bax and caspase 3/cleaved caspase 3 protein expression and increased Bcl2 protein expression	Bao, 2001; Liang et al., 2019; Yu et al., 2020a
		Ganoderic acid B	439.00	C_30_H_44_O_7_	APP/PS1 transgenic mice	Markedly reduced Bax and caspase 3/cleaved caspase 3 protein expression and increased Bcl2 protein expression	Bao, 2001; Liang et al., 2019; Yu et al., 2020a
Increase neurotrophic factor release	*Hericium erinaceus*	Erinacine A	433.26	C_25_H_36_O_6_	Mouse astroglial cells, 5-week-old rats	Acted to stimulate the synthesis of nerve growth factor NGF *in vitro*, increased the content of Catecholamine and NGF in the central nervous system of rats	(Kawagishi et al., 1994; Shimbo, Kawagishi, and Yokogoshi, 2005)
		Erinacine B	433.25	C_25_H_36_O_6_	Mouse astroglial cells	Had the activity of stimulating nerve growth factor NGF synthesis	Kawagishi et al. (1994)
		Erinacine D	478.29	C_27_H_42_O_7_	Mouse astroglial cells	Had the activity of stimulating nerve growth factor NGF synthesis	Kawagishi, (1996a)
		Erinacine E	432.25	C_25_H_36_O_6_	Mouse astroglial cells	Had the activity of stimulating nerve growth factor NGF synthesis	Kawagishi, (1996b)
		Erinacine F	433.25	C_25_H_36_O_6_	Mouse astroglial cells	Had the activity of stimulating nerve growth factor NGF synthesis	Kawagishi, (1996b)
		Erinacine G	—	C_25_H_36_O_8_	Mouse astroglial cells	Had the activity of stimulating nerve growth factor NGF synthesis	Kawagishi, (1996b)
		Erinacine H	471.24	C_25_H_35_O_7_Na	Rat astroglial cells	Had the activity of stimulating nerve growth factor NGF synthesis	Lee, (2001)
		Erinacine I	383.22	C_22_H_32_O_4_Na	Rat astroglial cells	Had the activity of stimulating nerve growth factor NGF synthesis	Lee, (2001)
		Hericenone C	571.40	C_35_H_54_O_6_	Mouse astroglial cells	Had the activity of stimulating nerve growth factor NGF synthesis	Kawagishi et al. (1991)
		Hericenone D	599.43	C_37_H_58_O_6_	Mouse astroglial cells	Had the activity of stimulating nerve growth factor NGF synthesis	Kawagishi et al. (1991)
		Hericenone E	599.43	C_37_H_54_O_6_	PC12 Cells	Enhanced NGF-induced axon growth mediated by ERK1/2 and PI3K/Akt	(Kawagishi et al., 1991; Phan et al., 2014)
	*Sarcodon scabrosus*	Scabronine A	482.51	C_22_H_34_O_6_	PC12 Cells	Promoted the release of various neurotrophin	(Ohta, 1998; Obara et al., 1999)
					1321N1 Cells		
		Scabronine B	512.26	C_33_H_36_O_5_	Rat astroglial cells	Had the activity of stimulating nerve growth factor NGF synthesis	Kita et al. (1998)
		Scabronine C	556.25	C_34_H_36_O_7_	Rat astroglial cells	Had the activity of stimulating nerve growth factor NGF synthesis	Kita et al. (1998)
		Scabronine D	452.22	C_27_H_32_O_6_	Rat astroglial cells	Had the activity of stimulating nerve growth factor NGF synthesis	Kita et al. (1998)
		Scabronine E	372.19	C_22_H_28_O_5_	Rat astroglial cells	Had the activity of stimulating nerve growth factor NGF synthesis	Kita et al. (1998)
		Scabronine F	330.18	C_20_H_26_O_4_	Rat astroglial cells	Had the activity of stimulating nerve growth factor NGF synthesis	Kita et al. (1998)
	*Dictyophora indusiata*	Dictyophorine A	232.15	C_15_H_20_O_2_	Rat astroglial cells	Promoted the synthesis of NGF	Kawagishi, (1997)
		Dictyophorine B	232.15	C_15_H_20_O_2_	Astroglial cells	Promoted the synthesis of NGF	Kawagishi, (1997)
	*Sarcodon cyrneus*	Cyrneines A	316.43	C_20_H_28_O_3_	PC12 Cells	Had the activity of stimulating nerve growth factor NGF synthesis	Marcotullio et al. (2006)
					1321N1 Cells		
		Cyrneines B	330.42	C_20_H_26_O_4_	PC12 Cells	Had the activity of stimulating nerve growth factor NGF synthesis	Marcotullio et al. (2006)
					1321N1 Cells		
	*Cyathus hookeri*	Cyahookerins A	405.26	C_24_H_38_O_5_	PC12 Cells	Enhanced axonal growth induced by NGF	Tang et al. (2019)
		Cyahookerins B	389.27	C_24_H_36_O_4_	PC12 Cells	Enhanced axonal growth induced by NGF	Tang et al. (2019)
		Cyahookerins C	408.27	C_23_H_34_O_5_	PC12 Cells	Enhanced axonal growth induced by NGF	Tang et al. (2019)
		Cyahookerins D	305.23	C_20_H_28_O_4_	PC12 Cells	Enhanced axonal growth induced by NGF	Tang et al. (2019)
		Cyahookerins E	401.27	C_23_H_38_O_4_	PC12 Cells	Enhanced axonal growth induced by NGF	Tang et al. (2019)
		Cyahookerins F	353.21	C_21_H_30_O_3_	PC12 Cells	Enhanced axonal growth induced by NGF	Tang et al. (2019)
Targeting ER stress	*Mycoleptodonoides aitchisonii*	3-hydroxymethyl-4-methylfuran-2(5H)-one	151.03	C_6_H_8_NaO_3_	Neuro2a cells	Inhibited the specific stress signal transduction of tunicamycin and reduce neuronal apoptosis caused by endoplasmic reticulum stress	Choi et al. (2009)
		1-hydroxy-3-pentanone	201.09	C_11_H_4_O_2_	Neuro2a cells	Inhibited the specific stress signal transduction of tunicamycin and reduce neuronal apoptosis caused by endoplasmic reticulum stress	Choi et al. (2009)
		3-(1′-hydroxyethyl)-4-methyldihydrofuran-2(3H)-one	167.06	C_7_H_12_NaO_3_	Neuro2a cells	Reduced neuronal apoptosis caused by endoplasmic reticulum stress	Choi et al. (2009)
		(3R*,4S*,1′S*)-3-hydroxyethyl-4-methyldihydrofuran-2(3H)-one	167.06	C_7_H_12_NaO_3_	Neuro2a cells	Reduced neuronal apoptosis caused by endoplasmic reticulum stress	Choi et al. (2009)
	*Termitomyces titanicus*	Termitomycamide B	459.30	C_26_H_39_NaNO_3_	Neuro2a cells	Inhibited the specific stress signal transduction, reduce endoplasmic reticulum stress	Choi et al. (2010)
		Termitomycamide E	422.30	C_28_H_40_N_2_O_2_	Neuro2a cells	Inhibited the specific stress signal transduction, reduce endoplasmic reticulum stress	Choi et al. (2010)
	*Stropharia rugosoannulata*	Strophasterol A	467.31	C_22_H_28_NaO_4_	Neuro2a cells	Reduced endoplasmic reticulum stress caused by Ca^2+^-ATPase inhibitor	Wu et al. (2012)
	*Leccinum extremiorientale*	Leccinine A	258.11	C_13_H_17_NO_3_	Neuro2a cells	Reduced endoplasmic reticulum stress caused by Ca^2+^-ATPase inhibitor	Choi et al. (2011)
*Targeting excitotoxicity*	*Dictyophora indusiata*	Dictyoquinazol A	313.12	C_17_H_17_O_4_N_2_	Mouse cortical cell	Protected primary cultured mouse cortical neurons from excitatory toxicity induced by glutamate and NMDA	ln-Kyoung (2002)
		Dictyoquinazol B	328.14	C_18_H_20_O_4_N_2_	Mouse cortical cell	Protected primary cultured mouse cortical neurons from excitatory toxicity induced by glutamate and NMDA	ln-Kyoung (2002)
		Dictyoquinazol C	342.12	C_18_H_18_O_5_N_2_	Mouse cortical cell	Protected primary cultured mouse cortical neurons from excitatory toxicity induced by glutamate and NMDA	ln-Kyoung (2002)
	*Amanita pantherina*	(2R), (1′R) and (2R), (1′S)-2-amino-3-(1,2-dicarboxyethylthio)propanoic acids	413.11	C_18_H_23_NO_8_	Bovine cerebral cortex membrane Rats	Inhibited 3H-glutamate receptor binding in bovine cerebral cortex membrane, displayed antagonistic activity against NMDA sensitive glutamate receptors in rat brain and spinal motor neurons	Fushiya 1 (1992)
Targeting mitochondrial dysfunction	*Ganoderma lucidum*	Ganoderterpene A	533.31	C_31_H_46_O_7_Na	BV-2 microglia cells	Inhibited the mitochondrial dysfunction caused by LPS	Kou et al. (2021a)

### 4.1 Targeting β-amyloid

The main neuropathological characteristic of AD involves irregular folding and aggregation of Aβ (Dhahri et al., 2022). The bioactive components derived from mushrooms that target Aβ proteins hold promise as potential therapeutic agents for AD. Both reduced Aβ degradation and enhanced Aβ production can lead to AD development (Hoshi, 2021a). Insulin-degrading enzymes (IDE) can degrade Aβ. APP is a transmembrane protein responsible for safeguarding the nervous system through the regulation of intracellular synaptic transmission and calcium balance, and can be cleaved to generate Aβ under the action of β-secretases and γ-secretases (Dobson, 2017; Lopez Sanchez, van Wijngaarden, and Trounce, 2019).

Erinacine A and erinacine S, cyathin diterpenoids isolated from the cultured mycelia of *H. erinaceus*, have been shown to reduce Aβ plaque growth and increase Aβ degradation by elevating IDE levels, thereby reducing the aggregation of Aβ in amyloid precursor protein/presenilin 1 (APP/PS1) double transgenic mice (Chen et al., 2016; Tzeng et al., 2018). Notably, erinacine A, but not erinacine S, effectively decreases the levels of insoluble amyloid β and the C-terminal fragment of APP (Tzeng et al., 2018).

**FIGURE 2:**
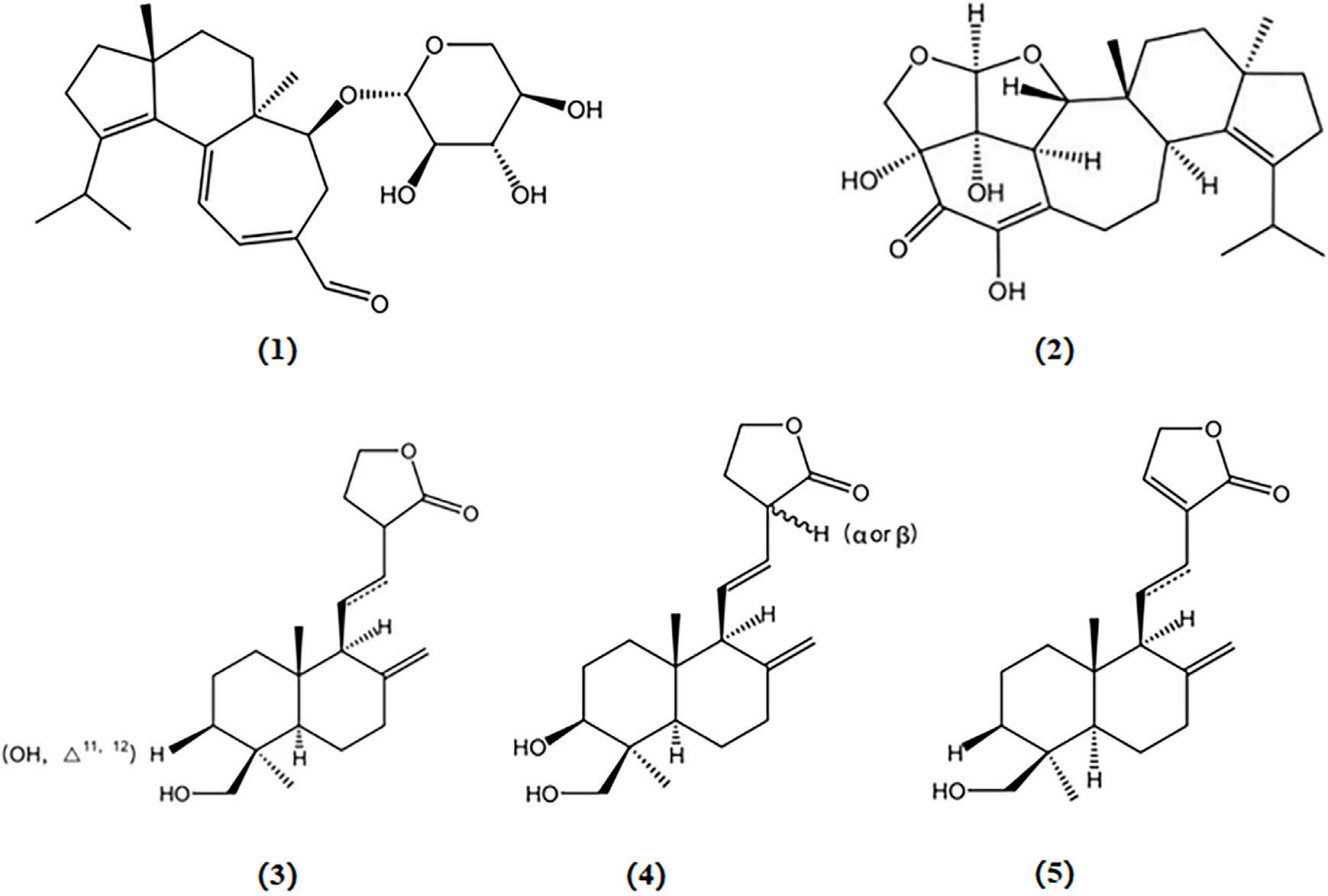
Chemical structures of mushroom bioactive components targeting β-amyloid. Structures of erinacine A (1), erinacine S (2) isolated from *H. erinaceus*; 13-epi-3β,19-dihydroxylabda-8 (17),11E-dien-16,15-olide (3), 3β,19-dihydroxylabda-8 (17),11E-dien-16,15-olide (4), and 19-hydroxylabda-8 (17)-en-16,15-olide (5) isolated from *A. camphorata*.

Primary cultures of neonatal cortical neurons from the cerebral cortex of Harlan Sprague Dawley rats, three labdane diterpenoids extracted from the *Antrodia camphorata* fruiting body, 13-epi-3β,19-dihydroxylabda-8 (17),11E-dien-16,15-oldie (A), 3β,19-dihydroxylabda-8 (17),11E-dien-16,15-olide (B), and 19-hydroxylabda-8 (17)-en-16,15-olide (C), were shown to mitigate Aβ-induced neurotoxicity and effectively safeguard neurons against Aβ-induced damage (Chen et al., 2006). The chemical structures of bioactive components from mushrooms that target β-amyloid for the treatment of AD are summarized in Figure 2.

### 4.2 Targeting tau

In the healthy brain, tau protein plays a vital role in preserving cellular integrity through the maintenance of microtubules. However, hyperphosphorylation of tau proteins results in self-interactions and disrupts their ability to bind to microtubules, thus causing NFTs formation (Dhakal et al., 2019). NFTs serve as significant pathophysiological hallmarks in the brain affected by AD. The formation of NFTs is closely linked to changes in synaptic function and neuronal plasticity (Grill and Cummings, 2010). Hyperphosphorylation of tau is a significant factor in the activation of astrocytes and microglia. This activation subsequently triggers the release of nuclear factor kappa B (NF-κB) and cytokines, leading to AD-related inflammation in the brain. Additionally, the release of inflammatory mediators such as interleukins (ILs) and NF-κB activates protein kinases within cells, further reinforcing tau hyperphosphorylation (Dhakal et al., 2019).

**FIGURE 3:**
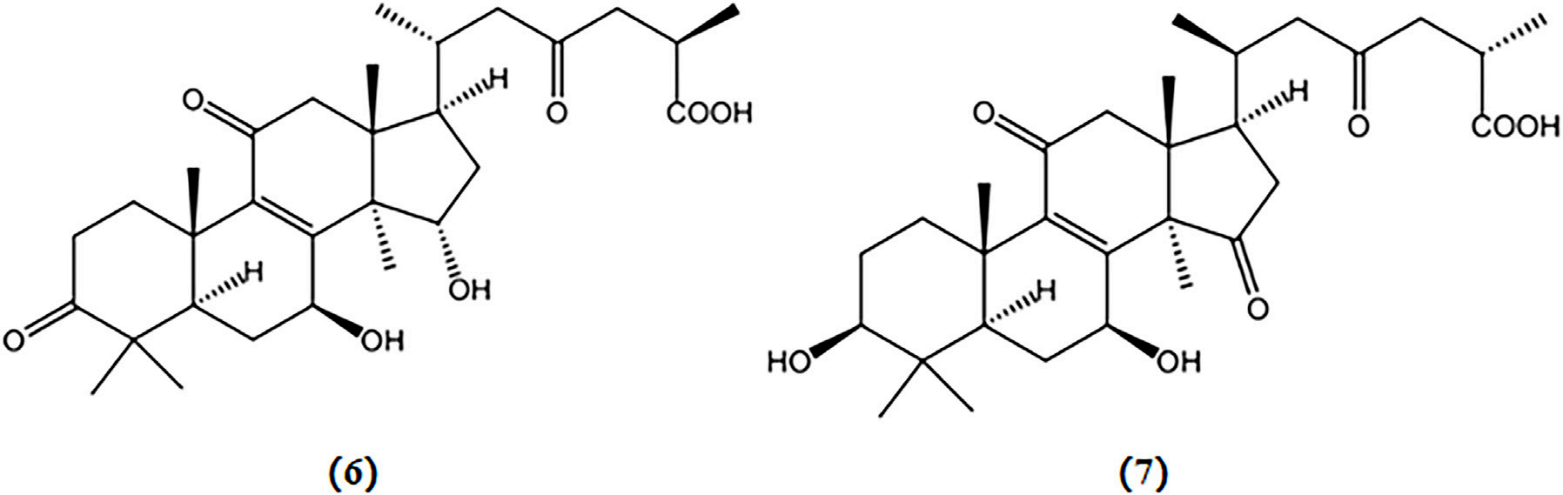
Chemical structures of mushroom bioactive components targeting tau. Structures of ganoderic acid A (6), and ganoderic acid B (7) isolated from *G. lucidum*.

Two triterpenoids extracted from *Ganoderma lucidum*, ganoderic acid A and ganoderic acid B, have been shown to reduce the number of NFT in the cytoplasm of APP/PS1 mice; and their structures are depicted in Figure 3 (Yu et al., 2020b).

### 4.3 Targeting cholinergic system

The levels of AChv at the synapse can be increased through the inhibition of AChE, leading to an enhancement of cholinergic activity in the brain (Anand and Singh, 2013). Existing AChE inhibitor drugs, such as galantamine, commonly prescribed to alleviate AD symptoms, may have associated gastrointestinal side effects, such as dizziness, headache, nausea, and vomiting. Therefore, there is a significant demand for anticholinesterase bioactive compounds derived from mushrooms with higher efficacy and fewer side effects to ameliorate AD.

**FIGURE 4:**
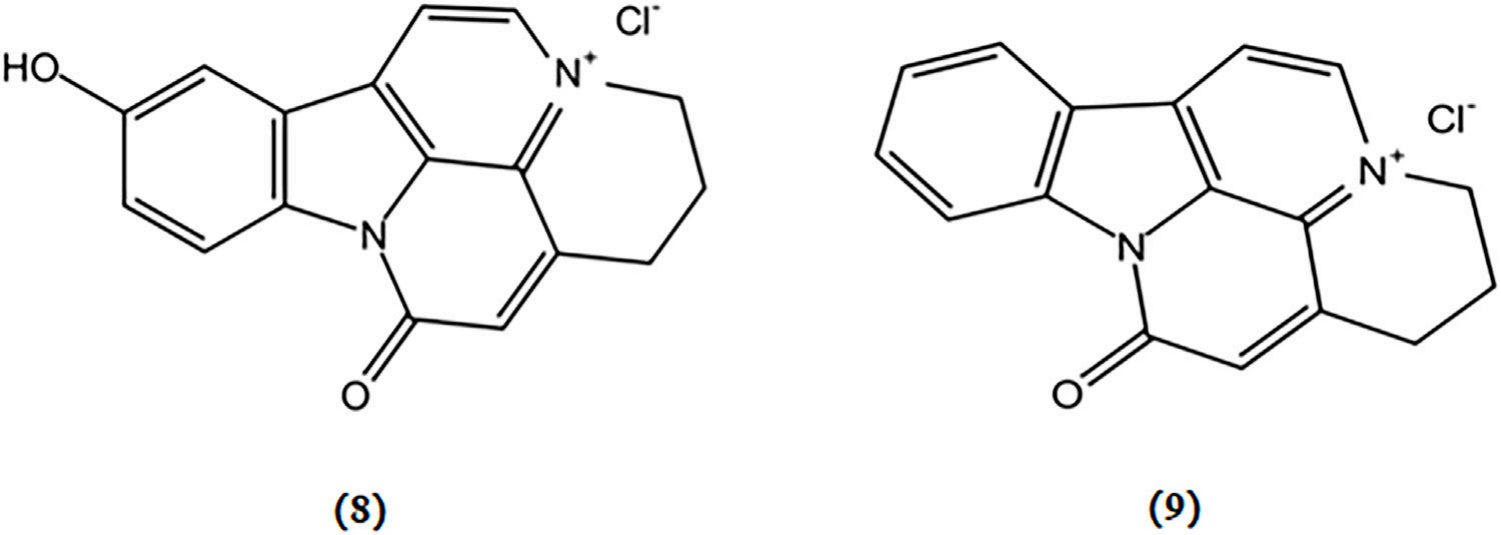
Chemical structures of mushroom bioactive components targeting cholinergic system. Structures of 10-hydroxy-infractopicrin (8), and infractopicrin (9) isolated from *C. infractus*.

10-hydroxy-infractopicrin and infractopicrin are alkaloids extracted from *Cortinarius infractus*, whose structures are shown in Figure 4. These two compounds demonstrated potent inhibitory activity against AChE and exhibited higher selectivity than galanthamine (Geissler et al., 2010). Hence, there is a promising prospect for developing mushroom-derived bioactive compounds with AChE inhibitory properties as novel therapeutic agents for the treatment of AD.

### 4.4 Targeting OS

*I.* *obliquus* is a well-known mushroom popular for its ability to exert anti-oxidative activity in neuronal cells (Phan et al., 2015). 3,4-dihydroxybenzalacetone, a phenolic ingredient extracted from *I. obliquus*, demonstrated protective effects via reducing the production of intracellular ROS in PC12 cells against OS induced by H_2_O_2_ (Nakajima et al., 2009b).

Antroquinonol, a ubiquinone derivative extracted from *A*. *camphorata*, can penetrate the blood-brain barrier. An APP transgenic mouse model demonstrated a reduction in hippocampal Aβ levels and the degree of astrogliosis, which may be mediated via the upregulation of the nuclear factor erythroid 2-related factor 2 (Nrf2) pathways (De Plano, 2023). After exposure to ROS, Nrf2 initiates the transcription of the genes responsible for antioxidant protection. These genes include those encoding heme oxygenases, glutathione peroxidases, superoxide dismutases, and peroxiredoxins. The resulting free radical-scavenging enzymes serve as robust antioxidant defense mechanisms, effectively countering oxidative damage (Lim et al., 2014). Therefore, antroquinonol shows potential as a complementary treatment for AD associated with OS.

**FIGURE 5:**
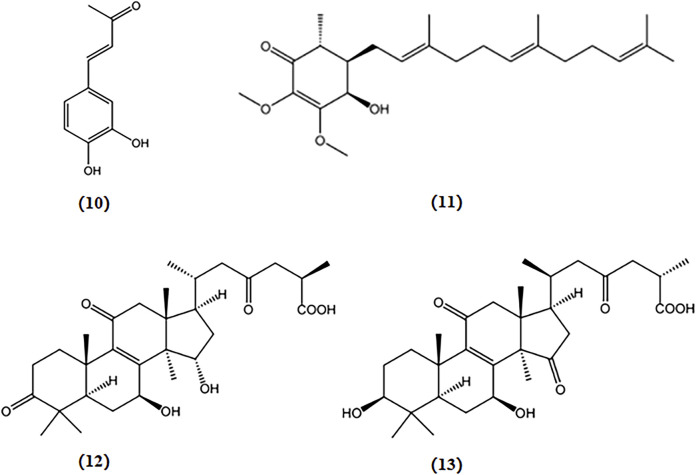
Chemical structures of mushroom bioactive components targeting OS. Structures of 3,4-dihydroxybenzalacetone (10) isolated from *I. obliquus*; antroquinonol (11) isolated from *A*. *camphorata*; ganoderic acid A (12), and ganoderic acid B (13) isolated from *G. lucidum.*

Ganoderic acid A and B, two triterpenoids derived from *G. lucidum*, restored the levels of antioxidant proteins Nrf2, NQO1, and HO-1, alleviating oxidative damage in the hippocampus of APP/PS1 mice, which potentially exerted a protective effect on AD (Yu et al., 2020b). The chemical structures of bioactive components from mushrooms that target OS for the treatment of AD are shown in Figure 5.

### 4.5 Targeting neuroinflammatory

Microglia, macrophage-like cells in the brain, are important for tissue repair and host defense in the CNS (Jeong et al., 2010). Under pathological conditions, activated microglia release pro-inflammatory and neurotoxic mediators such as pro-inflammatory cytokines and ROS (Meda et al., 1995). The excessive production of these inflammatory mediators has been implicated in the pathogenesis of AD. Consequently, inhibiting excessive activation of microglia, decreasing the release of pro-inflammatory factors, and modulating relevant pathways could alleviate the severity of AD.

Cordycepin, a nucleoside analog similar to adenosine in structure, was initially derived from the fermented broth of Cordyceps militaris. Cordycepin significantly suppressed the release of inflammatory mediators such as IL-1β, TNF-α, PGE2, NO, iNOS, and COX-2 in murine BV2 microglia stimulated with LPS by inhibiting the NF-κB, mitogen-activated protein kinase (MAPK), and protein kinase B (Akt) pathways (Jeong et al., 2010; Peng et al., 2015). Cordycepin exhibits the ability to restore neuronal death caused by excessive microglial activation and rescue impairments of neural development in cultured hippocampal neurons induced by inflammation (Peng et al., 2015). These findings indicate that cordycepin is a promising alternative therapeutic strategy for AD treatment.

**FIGURE 6:**
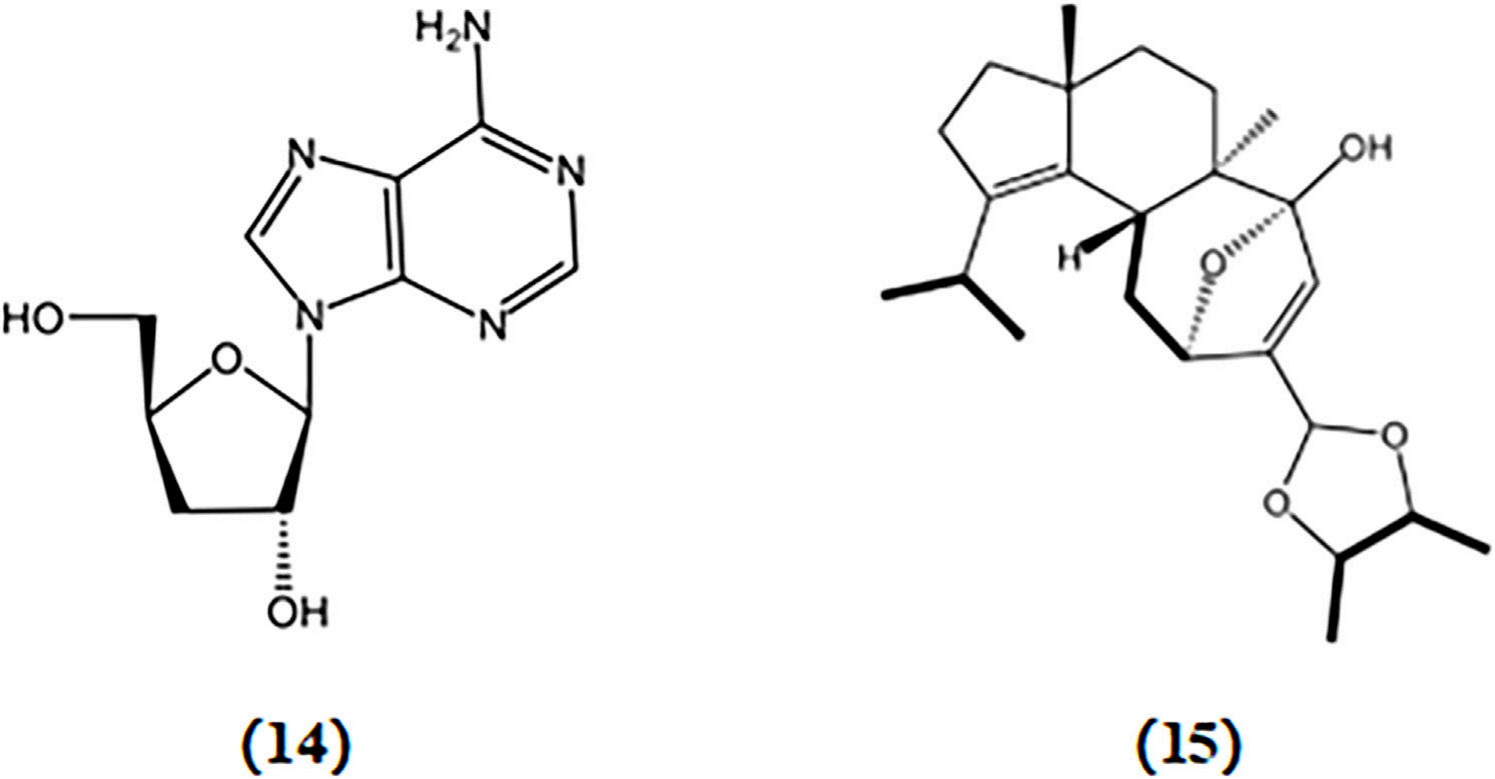
Chemical structures of mushroom bioactive components targeting neuroinflammatory. Structures of cordycepin (14) isolated from *C*. *militaris*; cyahookerin B (15) isolated from *C*. *hookeri*.

Cyahookerin B, a cyathane diterpenoid, was extracted from a liquid culture of *Cyathus hookeri*. This compound exhibited notable inhibition of NO production in LPS-activated BV-2 microglial cells, indicating its potential to reduce neuroinflammation associated with AD (Tang et al., 2019). The chemical structures of bioactive components from mushrooms that target neuroinflammatory for the treatment of AD are shown in Figure 6.

### 4.6 Targeting anti-apoptotic

The Bcl-2 family, which consists of Bcl-2 and Bax, plays a vital role in regulating neuronal cell apoptosis. Enhanced expression of Bax promotes neuronal apoptosis, whereas enhanced expression of Bcl-2 suppresses apoptosis (Peña-Blanco and García-Sáez, 2018a). Caspase-3, which is recognized as a vital executor of apoptosis, serves as a death protease. It induces cell death by enzymatically cleaving apoptosis inhibitors and related DNA repair molecules (Zhang, Wang, et al., 2022a).

3,4-dihydroxybenzalacetone, a phenolic ingredient extracted from *I. obliquus*, inhibits effects on neuronal apoptosis by inhibiting Bax and p38-MAPK in PC12 cells (Nakajima et al., 2009b). Thus, 3,4-dihydroxybenzalacetone may alleviate AD progression.

**FIGURE 7:**
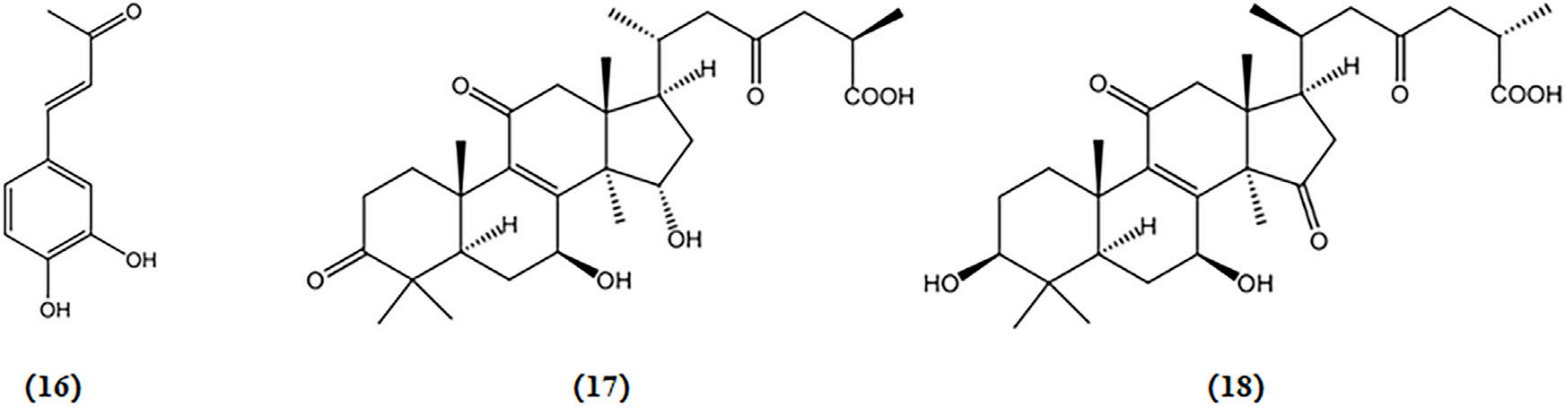
Chemical structures of mushroom bioactive components targeting anti-apoptotic. Structures of 3,4-dihydroxybenzalacetone (16) isolated from *I. obliquus*; ganoderic acid A (17) and ganoderic acid B (18) isolated from *G. lucidum*.

Ganoderic acid A and B, triterpenoids derived from *G. lucidum*, demonstrate a protective effect in APP/PS1 transgenic mice by significantly decreasing the protein expression of Bax and caspase 3/cleaved caspase 3, while increasing the protein expression of Bcl2 in hippocampal tissues (Yu et al., 2020b). The chemical structures of bioactive components from mushrooms that target anti-apoptotic activities for the treatment of AD are shown in Figure 7.

### 4.7 Targeting NTFs

*H. erinaceus* has garnered considerable attention owing to its therapeutic activity in promoting brain and nerve health. Various compounds derived from *H. erinaceus* such as erinacines and hericenones, have been investigated and reported for their ability to induce NGF expression (Li et al., 2018). Erinacines are cyathin diterpenoids that possess bioactive properties that stimulate NGF synthesis. These compounds have potential therapeutic applications in the treatment of AD (Apfel and Kessler, 1996). Since the fruiting body was found to lack erinacine, the optimal approach was to increase the production of erinacine in *H. erinaceus* mycelia through submerged fermentation while maintaining precise control over the culture conditions. Fifteen erinacines (erinacines A-K and P-S) have been identified thus far, and subsequent studies have revealed that eight of these (erinacines A-B, D-I) exhibit neuroprotective effects through the enhancement of NGF release (Li et al., 2018). The primary representative of the erinacine A group is erinacine A, which has been found to effectively enhance the NGF content in both *in vitro* and *in vivo* studies. Erinacine A stimulates NGF synthesis in mouse astroglial cells (Kawagishi et al., 1994). In addition, 8 mg/kg erinacine A increased NGF content in various brain regions, such as the hippocampus and locus coeruleus of rats (Shimbo, Kawagishi, and Yokogoshi, 2005). Hericenones C-E, another group of compounds extracted from *H. erinaceus*, have been found to possess stimulating properties for NGF synthesis in mouse astroglial cells (Kawagishi et al., 1991). Studies indicate that among these hericenones, hericenone E exhibits the highest activity, which can enhance NGF-induced neuritogenesis through the regulation of the PI3K/Akt and MEK/ERK signaling pathways in PC12 cells (Phan et al., 2014).

*Sarcodon scabrosus*, a bitter mushroom belonging to the family *Hydnaceae*, is a basidiomycete fungus. Compounds derived from *S. scabrosus* such as scabronine, have been shown to enhance NGF synthesis in human nerve cells. This suggests their potential as therapeutic agents for AD treatment (Shi et al., 2011). Scabronines are a class of novel diterpenoid xylosides with the same carbon skeleton, isolated from *S. scabrosus*. Scabronine B-F has been reported to demonstrate significantly stimulates NGF synthesis in rat astroglial cells (Kita et al., 1998). The compound scabronine A was found to enhance the gene expression of NGF and stimulate the secretion of NGF in 1321N1 human astrocytoma cells (Obara et al., 1999).

*Dictyophora indusiata* is a saprophytic fungus from the *Phallaceae* family that is widely consumed and celebrated as one of the most popular edible mushrooms in Asian countries, particularly China. It is highly regarded for its delightful taste, appealing appearance, and remarkable nutritional properties (Wu et al., 2012). Dictyophorine A and B derived from *D. indusiata* were also found to enhance the synthesis of NGF in rat astroglial cells (Lim et al., 2023).

Two novel diterpenoids, cyrneine A and cyrneine B, were extracted from *Sarcodon cyrneus* and were found to induce NGF synthesis in a neuronal differentiation model using rat PC12 cells (Marcotullio et al., 2006).

**FIGURE 8:**
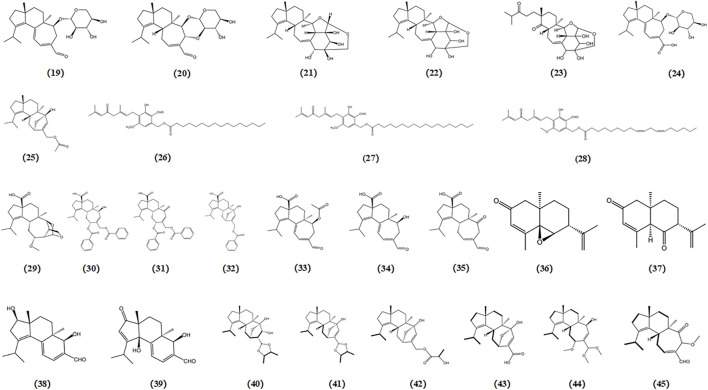
Chemical structures of mushroom bioactive components targeting NTFs. Structures of erinacine A (19), erinacine B (20), erinacine D-I (21-26), hericenone C-E (27-29) isolated from *H. erinaceus*; scabronine A-F (30-35) isolated from *S*. *scabrosus*; dictyophorine A (36), dictyophorine B (37) isolated from *D*. *indusiata*; cyrneines A (38), cyrneines B (39) isolated from *S. cyrneus*; cyahookerins A-F (40-45) isolated from *C*. *hookeri*.

Six cyathane diterpenoids, cyahookerins A-F, derived from the liquid culture of *C. hookeri*, have been demonstrated to enhance neurite outgrowth induced by NGF in PC-12 cells (Tang et al., 2019). The chemical structures of bioactive components from mushrooms that target NTFs for the treatment of AD are shown in Figure 8.

### 4.8 Targeting ER stress

Under specific pathological stress conditions, disturbances in ER homeostasis can occur, leading to depletion of intracellular calcium stores, loss of the oxidative environment within the ER, and accumulation of misfolded proteins in the ER (Kaufman, 1999). This condition, known as ER stress, can lead to apoptosis of neural cells in the brain and is considered a significant factor in the development of AD (Choi et al., 2009).

Tunicamycin, an inhibitor of N-glycosylation of glycoproteins, induces ER stress by inhibiting dolichol pyrophosphate-mediated glycosylation of the asparaginyl residues of glycoproteins (Choi et al., 2010; Wu et al., 2012). 3-hydroxymethyl-4-methylfuran-2(5H)-one (A), 1-hydroxy-3-pentanone (B), 3-(1′-hydroxyethyl)-4-methyldihydrofuran-2(3H)-one (C), and (3R*,4S*,1′S*)-3-hydroxyethyl-4-methyldihydrofuran-2(3H)-one (D) were four compounds purified from *Mycoleptodonoides aitchisonii*. Studies have reported that compounds A and B exert inhibitory effects on the tunicamycin-specific stress pathway, whereas compounds C and D protect cells by modulating ER stress signaling in neuro2a cells (Choi et al., 2009). Two fatty acid amides derived from *Termitomyces titanicus*, termitomycamide B and E, have also been found to exhibit inhibitory effects on stress signal transduction and mitigate ER stress induced by tunicamycin in Neuro2a cells (Choi et al., 2010).

**FIGURE 9:**
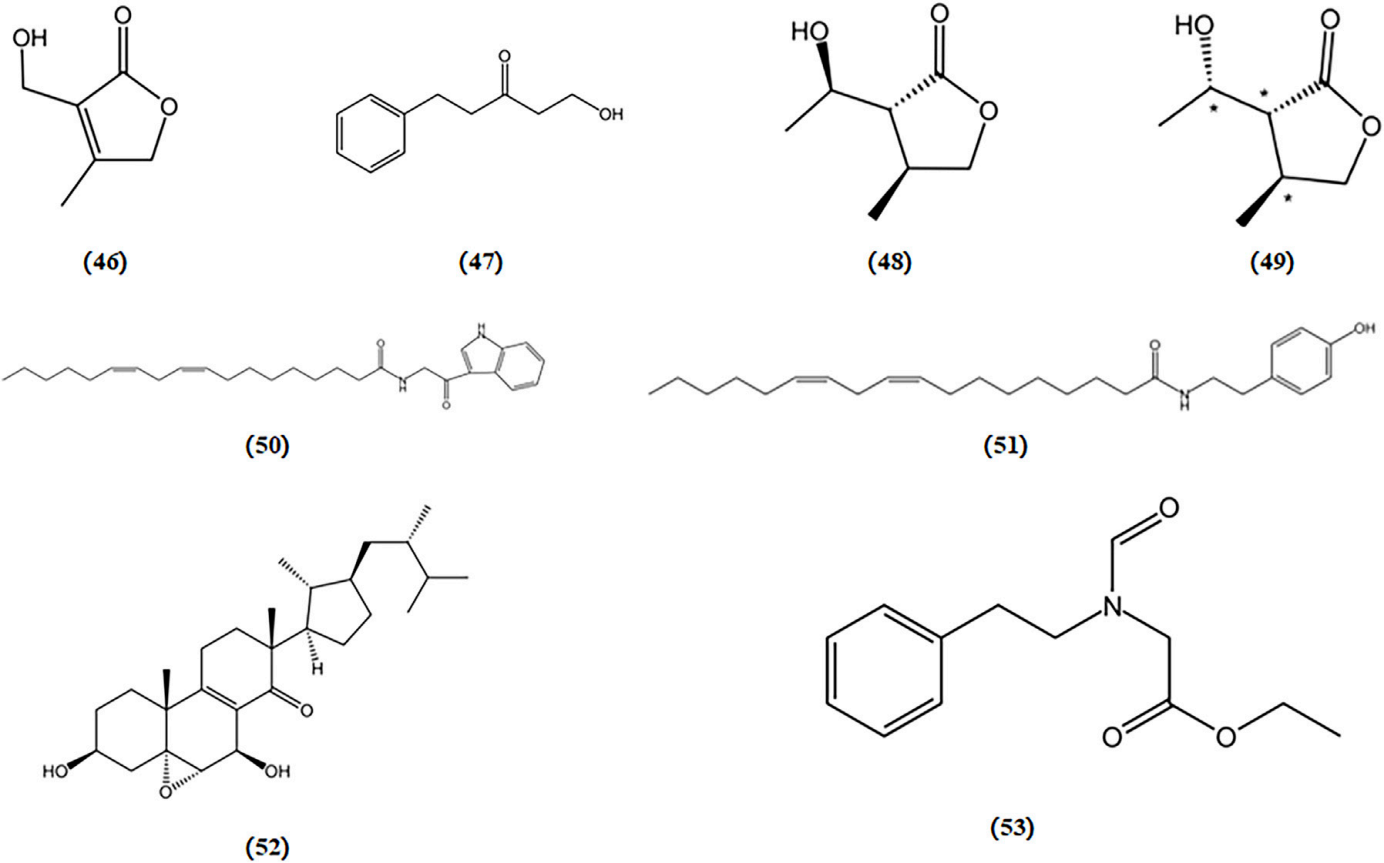
Chemical structures of mushroom bioactive components targeting ER stress. Structures of 3-hydroxymethyl-4-methylfuran-2(5H)-one (46), 1-hydroxy-3-pentanone (47), 3-(1′-hydroxyethyl)-4-methyldihydrofuran-2(3H)-one (48), (3R*,4S*,1S*)-3-hydroxyethyl-4-methyldihydrofuran-2(3H)-one (49) isolated from *M. aitchisonii*; termitomycamide B (50), termitomycamide E (51) isolated from *T. titanicus*; strophasterol A (52) isolated from *S*. *rugosoannulata*; leccinine A (53) isolated from *L. extremiorientale.*

Thapsigargin, an inhibitor of sarcoplasmic/ER Ca^2+^-ATPase, induces ER stress by disrupting Ca^2+^ homeostasis in the ER. In a protective activity assessment against thapsigargin-induced cell death dependent on ER stress, both strophasterol A (a novel steroid isolated from *Stropharia rugosoannulata*) and leccinine A (an ethyl 2-(N-phenethylformamido) acetate isolated from *Leccinum extremiorientale*) exhibited the ability to safeguard neuronal cells by alleviating ER stress triggered by a Ca^2+^-ATPase inhibitor (Choi et al., 2011; Wu et al., 2012). The chemical structures of bioactive components from mushrooms that target ER stress for the treatment of AD are shown in Figure 9.

### 4.9 Targeting excitotoxicity

The overactivity of glutamate receptors has been linked to the progression of neuronal cell death. NMDA and α-amino-3-hydroxy-5-methyl-4-isoxazolepropionic acid (AMPA) receptors have been identified as significant factors contributing to neuronal death in the brain. Consequently, significant efforts have been devoted to discovering compounds that can inhibit these receptors as a potential therapeutic approach for treating AD.

Three novel quinazoline compounds, known as dictyoquinazols A, B, and C, were extracted from the mushroom *D. indusiata*. Their ability to shield neuronal cells from excitotoxicity was evaluated by observing primary cultured mouse cortical neurons treated with excitatory neurotoxins such as glutamate, AMPA, NMDA, and kainate. The findings indicated that dictyoquinazols A, B, and C act as glutamate receptor antagonists, particularly NMDA receptor antagonists, rather than antioxidants, in protecting neuronal cells against glutamate-induced damage (ln-Kyoung Lee 1 2002).

**FIGURE 10:**
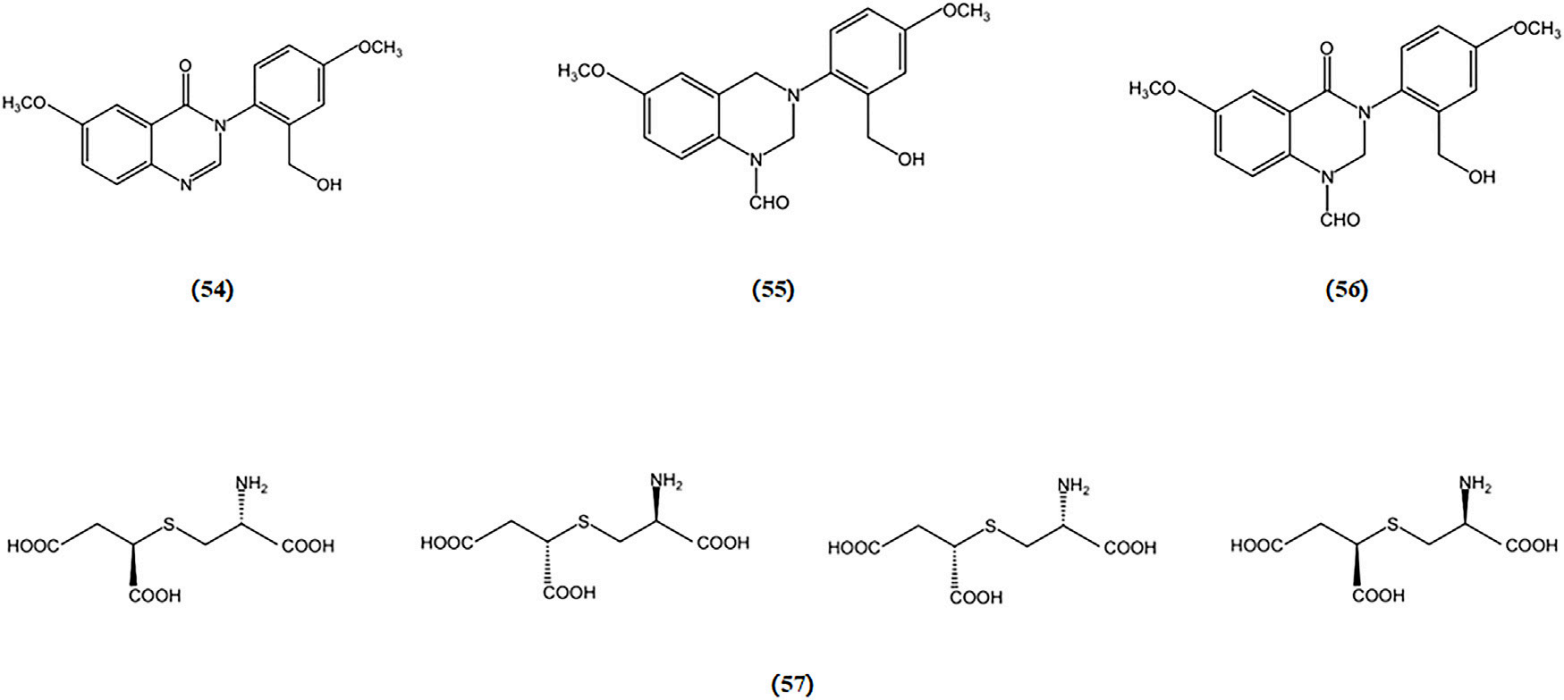
Chemical structures of mushroom bioactive components targeting excitotoxicity. Structures of dictyoquinazol A (54), dictyoquinazol B (55), dictyoquinazol C (56) isolated from *D. indusiata* and (2R), (1′R) and (2R), (1′S)-2-amino-3-(1,2-dicarboxyethylthio) propanoic acids (57) isolated from *A*. *pantherina*.

In another study, a mixture of diastereoisomers of 2-amino-3-(1,2-dicarboxyethylthio) propanoic acids was isolated from the *Amanita pantherina*. This mixture was separated, and the absolute configurations of its components were identified as (2R), (1′R), and (2R), (1′S). These components exhibited antagonistic activity against NMDA-sensitive glutamate receptors in rat brain and spinal motoneurons (S Fushiya 1 1992). The chemical structures of bioactive components from mushrooms that target excitotoxicity for the treatment of AD are shown in Figure 10.

### 4.10 Targeting mitochondrial dysfunction

**FIGURE 11:**
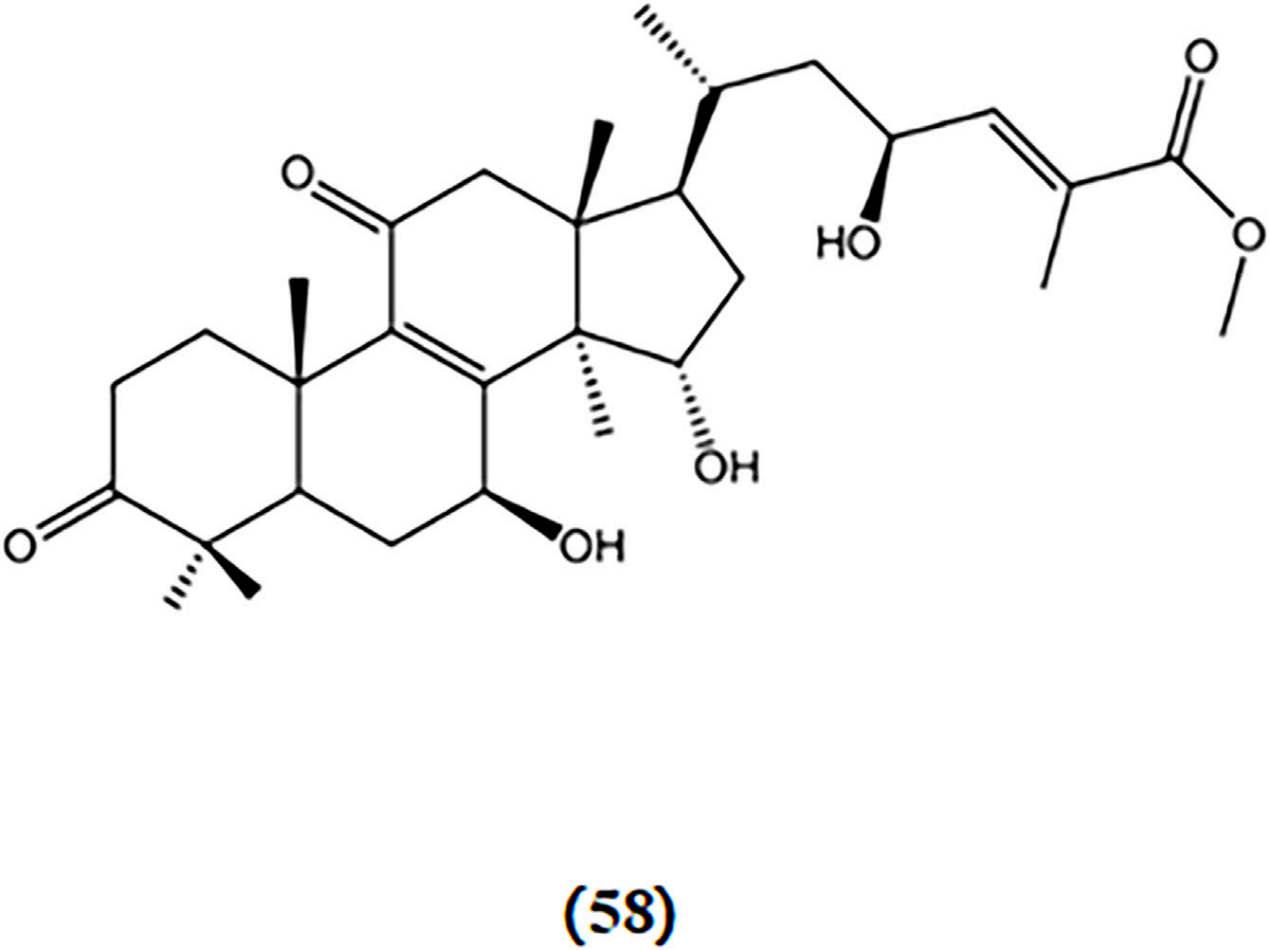
Chemical structures of mushroom bioactive components targeting mitochondrial dysfunction. Structures of ganoderterpene A (62) isolated from *G. lucidum*.

The deficiency in mitochondrial energy metabolism has long been regarded as one of the pathological foundations of AD (Golpich et al., 2017). Mitochondria are where energy substances are ultimately oxidized to release energy. LPS can induce mitochondrial damage. Ganoderterpene A, a new lanostane-type triterpene compound isolated from the fruiting bodies of the edible mushroom *G. lucidum*, was studied for its effect on the MMP induced by LPS in BV-2 microglia cells. The results demonstrated that ganoderterpene A significantly mitigated the LPS-induced decrease in MMP in BV-2 microglia cells, which means that ganoderterpene A could notably inhibit the mitochondrial dysfunction caused by LPS (Kou et al., 2021b). The chemical structures of Ganoderterpene A is shown in Figure 11.

## 5 Mushroom-derived bioactive components with definite structures in clinical study of AD

Mushroom-derived bioactive components have gained widespread acceptance as dietary supplements, believed to enhance health through various mechanisms, including aiding with neurasthenia, anemia, or insomnia and even boosting memory function (Yadav et al., 2020). Although preclinical trials have demonstrated the potential of mushroom-derived bioactive components with definite structures to combat AD, published clinical studies are scarce. Several exceptions are clinical trials, in which patients were administered mushrooms or their components for AD treatment.

In a double-blind, parallel-group, placebo-controlled trial involving 30 patients with mild cognitive impairment, 1,000 mg of 96% pure *H. erinaceus* mushroom powder was administered thrice daily for 16 weeks. A follow-up assessment was conducted for 4 weeks post-treatment, and cognitive function was measured using a revised version of the Hasegawa Dementia Scale. These findings indicated that the treatment group experienced an enhancement in cognitive function compared to the placebo group. Notably, there were no significant adverse effects, with only one participant withdrawing owing to stomach pain (Mori et al., 2009).

Furthermore, another investigation was carried out to evaluate the potential of *H. erinaceus* mycelia enriched with erinacine A against early AD. This randomized, double-blind, placebo-controlled pilot study spanned a period of 49 weeks. During this trial, the patients were administered three capsules daily, each containing 350 mg of *H. erinaceus* mycelium and 5 mg/g of erinacine A. The findings indicated an improvement in the instrumental activities of daily living score, contrast sensitivity, and Mini-Mental State Examination score compared with the placebo group. In terms of AD-specific markers, the experimental group exhibited reduced APOE4 expression and lower levels of Aβ_1-40_, along with increased concentrations of brain-derived neurotrophic factor. Four participants discontinued participation because of skin rash, nausea, and abdominal discomfort (Li et al., 2020b).

Although some clinical and laboratory evidence supports the safety and effectiveness of mushroom-derived bioactive components, the interpretation of this evidence is complicated by statistical heterogeneity in study outcomes, differences in patient populations, extraction methods, and dosage regimens. It is clear that high-quality randomized, double-blind, placebo-controlled studies are required to fully integrate these valuable medicines into global healthcare facilities. Through this review, we aim to foster an interest in discovering new compounds with definite structures from mushrooms that could aid in AD, with the hope that such advancements will contribute to the ultimate goal of creating efficacious treatments for AD.

## 6 Limitations and future directions

This review explores the potential use of mushroom bioactive components with well-defined structures for AD treatment. Nevertheless, certain unresolved challenges and limitations, particularly regarding bioavailability, safety, pharmacokinetics, dose efficiency, administration route, delivery system, and clinical status, remain in the application of these mushroom bioactive components as therapeutic interventions for AD. (1) In terms of safety, while numerous mushrooms are considered edible, caution must be exercised when utilizing them in clinical trials, as their potential adverse effects have not been well established. In addition, it is crucial to consider the potential interactions between the bioactive components derived from mushrooms and clinically prescribed drugs. Although some mushroom bioactive components are currently regarded as supplements, it is important to recognize that they are not intended to replace prescription drugs. Few studies have compared the therapeutic effects and safety profiles of mushroom-derived bioactive components with current pharmacological treatments for AD. (2) Currently, reports of bioactive components with well-defined structures from mushrooms in AD treatment are scarce. Therefore, there is a need to identify new bioactive components in different mushroom species. (3) Undoubtedly, mushroom bioactive components have significant benefits and potential for managing AD. Because of the complicated and variable structures and properties of mushroom-derived bioactive components, the actual biochemical interactions and mechanisms of action are inconclusive and require further in-depth research in the future. In addition, the limited applicability of these components stems from a lack of comprehensive understanding of their pharmacodynamics and pharmacokinetics. (4) In numerous cases, the bioavailability of these compounds in the CNS is restricted by challenges such as rapid metabolism, low absorption in the gastrointestinal tract, and impermeability permeability across the blood-brain barrier (Pandareesh, Mythri, and Bharath, 2015; Dhakal et al., 2019). The processing and initial metabolism of bioactive components derived from mushrooms occur at different levels, including in the liver, stomach, large intestine, small intestine, and circulatory system, which can induce significant alterations in the quantity, structure, and biological activity of these components. Even more crucial in AD is the requirement for these mushroom bioactive components to cross the blood–brain barrier from the bloodstream to the brain tissue to reach their targets. This process is influenced by the lipophilicity of the components (Dhakal et al., 2019). Hence, future studies should focus on enhancing the bioavailability of these mushroom compounds in the human body, particularly in the brain, to maximize their effects on AD treatment. (5) The anti-AD effects of bioactive components from mushrooms have primarily been demonstrated in cellular and animal models. The translation of these findings into human trials still poses significant challenges. To further substantiate their therapeutic benefits in AD patients, it is crucial to conduct large-scale randomized clinical studies on these mushroom-derived bioactive components.

## 7 Conclusion

This review provides a comprehensive summary of the latest findings on the therapeutic effects of mushroom bioactive compounds with well-defined structures in the treatment of AD and emphasizes the significant roles and molecular mechanisms of these compounds in treating AD. These findings provide potential opportunities for utilizing mushroom bioactive compounds as promising therapeutic agents for AD treatment.

## Abbreviations

AMPA, α-amino-3-hydroxy-5-methyl-4-isoxazolepropionic acid; ACh, Acetylcholine; AChE, Acetylcholinesterase; AChEIs, Acetylcholinesterase inhibitors; AD, Alzheimer’s disease; Aβ, Amyloid beta; A. pantherina, Amanita pantherina; APP, Amyloid precursor protein; APP/PS1, Amyloid precursor protein/presenilin 1; A. camphorata, Antrodia camphorata; Bcl-2, B-cell lymphoma-2; Bax, Bcl-2-associated x; BDNF, Brain-derived neurotrophic factor; CNS, Central nervous system; ChAT, Choline acetyltransferase; C. militaris, Cordyceps militaris; C. infractus, Cortinarius infractus; C. hookeri, Cyathus hookeri; Cyt-C, Cytochrome C; D. indusiata, Dictyophora indusiata; ER, Endoplasmic reticulum; G. lucidum, Ganoderma lucidum; GDNF, Glial cell-derived neurotrophic factor; H. erinaceus, Hericium erinaceus; IDE, Insulin degrading enzyme; ILs, Interleukins; iNOS, Inducible nitric oxide synthase; I. obliquus, Inonotus obliquus; ILs, Interleukins; L. extremiorientale, Leccinum extremiorientale; LPSLipopolysaccharide; MMP, Mitochondrial membrane potential; MAPK, Mitogen activated protein kinase; M. aitchisonii, Mycoleptodonoides aitchisonii; NFTs, Neurofibrillary tangles; NTFs, Neurotrophic factors; NGF, Nerve growth factor; NO, Nitric oxide; NMDA, N-methyl-D-aspartate; Nrf2, Nuclear factor erythroid 2-related factor 2; NF-κB, Nuclear factor kappa B; OS, Oxidative stress; Akt, Protein kinase B; ROS, Reactive oxygen species; S. cyrneus, Sarcodon cyrneus; S. scabrosus, Sarcodon scabrosus; S. rugosoannulata, Stropharia rugosoannulata; T. titanicus, Termitomyces titanicus.
