# Supplementary material for: Mushroom-derived bioactive components with definite structures in alleviating the pathogenesis of Alzheimer’s disease
Source: Front Pharmacol. 2024 May 21;15:1373660. doi: 10.3389/fphar.2024.1373660 (PMC11148366; doi:10.3389/fphar.2024.1373660)
Supplement: Supplementary file 1 [file DataSheet1.docx]

Supplementary Material

# Supplementary Figures and Tables

## Supplementary Figures


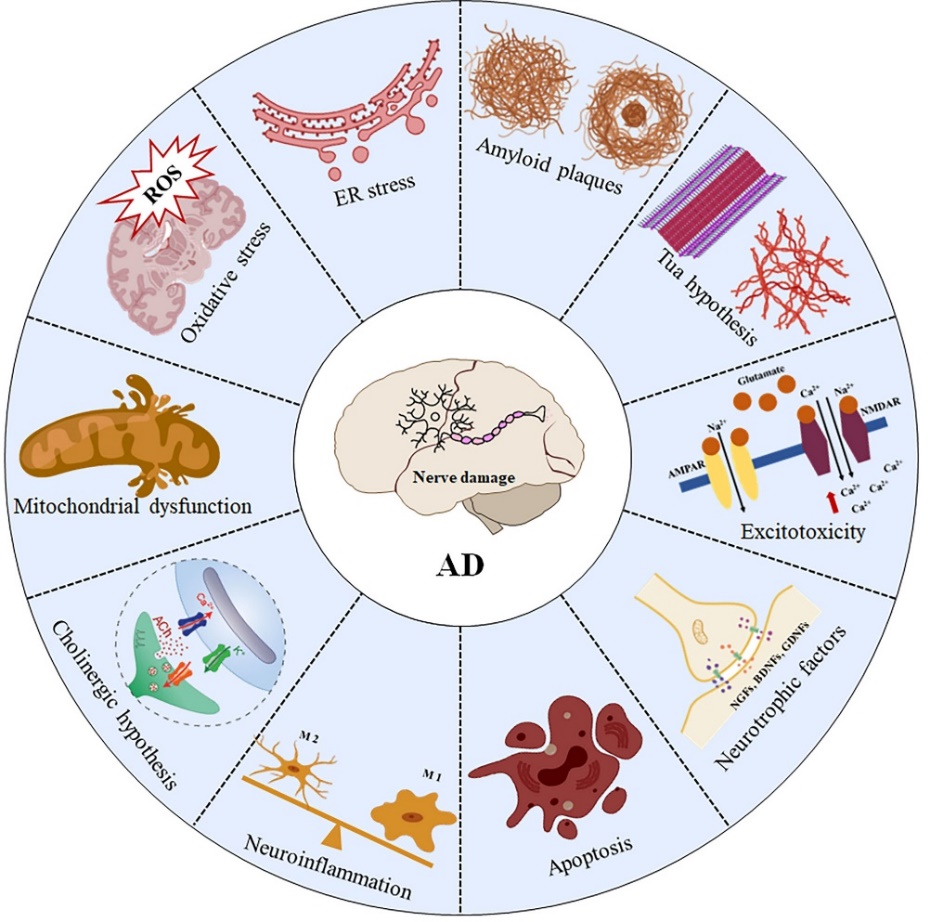


**Supplementary Figure 1.** Overview of the main mechanisms involved in pathogenesis of AD.

## Supplementary Tables

**Table S1.** Effects and side effects of medications for the treatment of AD.

| **Types** | **Drug** | **Effects** | **Adverse effect** | **Clinical study** | **Reference** |
| --- | --- | --- | --- | --- | --- |
| Glutamate regulator | Memantine | Prevent neuronal necrosis caused by glutamate calcium neurotoxicity, block excessive NMDA receptor activity | Dizziness, headache, constipation, drowsiness, hypertension | Listed | (1, 2) |
| Acetylcholinesterase inhibitors | Donepezil | Increase cortical α7-nAChR and synaptic acetylcholine levels | Diarrhea, nausea, headache | Listed | (3, 4) |
|  | Rivastigmine | Increase levels of acetylcholine chemicals in the brain | Weight loss, excessive salivation, nausea and vomiting, mental retardation, abdominal pain | Listed | (5, 6) |
|  | Galantamine | Competitively inhibit AChE, modulate nicotinic receptors by allosterically potentiating submaximal nicotinic responses to ACh | Vomiting, nausea, headache, diarrhoea, decreased appetite, dizziness | Listed | (7, 8) |
| Drugs directed at β1 amyloid | Aducanumab | Reducte amyloid β plaque | Amyloid-related imaging abnormalities | Listed | (9) |
|  | Verubecestat | Decrease the concentrations of cerebral Aβ, reduce the formation of dendritic spines | May cause insufficient pigmentation | Phase III clinical trial | (10) |
| Calcium channel blockers | Nimodipine | Help to conduct Ion channel inflow through calcium in plasma membrane, leading to excitotoxic neurodegeneration | May be a risk of cartilage toxicity | Listed | (11, 12) |

References

1. Ong WY, Tanaka K, Dawe GS, Ittner LM, Farooqui AA. Slow excitotoxicity in Alzheimer's disease. J Alzheimers Dis. 2013;35(4):643-68. Epub 2013/03/14. doi:10.3233/JAD-121990. Cited in: Pubmed; PMID 23481689.

2. Marum RJv. Update on the use of memantine in Alzheimer’s disease. 2009 05-20. doi:10.2147/ndt.s4048.

3. R S Doody 1 DSG, B Gordon , C A Perdomo , R D Pratt ,. Open-label, multicenter, phase 3 extension study of the safety and efficacy of donepezil in patients with Alzheimer disease. Archives of Neurology. 2001 03-20. doi:10.1001/archneur.58.3.427.

4. Richard T Reid MNS. Effects of donepezil treatment on rat nicotinic acetylcholine receptor levels in vivo and in vitro. Journal of Alzheimer’s Disease. 2004 02-06. doi:DOI: 10.3233/jad-2003-5602.

5. F Forette 1 RA, G Gharabawi. A phase II study in patients with Alzheimer's disease to assess the preliminary efficacy and maximum tolerated dose of rivastigmine (Exelon). European Journal of Neurology. 1999 06-11. doi:10.1046/j.1468-1331.1999.640423.x.

6. Birks JS, Grimley Evans J. Rivastigmine for Alzheimer's disease. Cochrane Database Syst Rev. 2015 Apr 10;(4):CD001191. Epub 2015/04/11. doi:10.1002/14651858.CD001191.pub3. Cited in: Pubmed; PMID 25858345.

7. Wilkinson D, Murray J. Galantamine: a randomized, double-blind, dose comparison in patients with Alzheimer's disease. Int J Geriatr Psychiatry. 2001 Sep;16(9):852-7. Epub 2001/09/26. doi:10.1002/gps.409. Cited in: Pubmed; PMID 11571763.

8. Seltzer B. Galantamine-ER for the treatment of mild-to-moderate Alzheimer’s disease. 2009 12-01. doi:10.2147/cia.s4819.

9. The Lancet N. A contentious FDA ruling for Alzheimer's disease. Lancet Neurol. 2021 Aug;20(8):585. Epub 2021/07/09. doi:10.1016/S1474-4422(21)00215-5. Cited in: Pubmed; PMID 34237273.

10. Matthew E. Kennedy AWS. The BACE1 inhibitor verubecestat (MK-8931) reduces CNS β-amyloid in animal models and in Alzheimer’s disease patients. Science Translational Medicine. 2016 11-02. doi:DOI:10.1126/scitranslmed.aad9704.

11. Nimmrich V, Eckert A. Calcium channel blockers and dementia. Br J Pharmacol. 2013 Jul;169(6):1203-10. Epub 2013/05/04. doi:10.1111/bph.12240. Cited in: Pubmed; PMID 23638877.

12. Kaplan N, Yilmaz I, Karaarslan N, Kaya YE, Sirin DY, Ozbek H. Does Nimodipine, a Selective Calcium Channel Blocker, Impair Chondrocyte Proliferation or Damage Extracellular Matrix Structures? Curr Pharm Biotechnol. 2019;20(6):517-524. Epub 2019/05/07. doi:10.2174/1389201020666190506124548. Cited in: Pubmed; PMID 31057106.
